# Skeletal Muscle DNA Damage Precedes Spinal Motor Neuron DNA Damage in a Mouse Model of Spinal Muscular Atrophy (SMA)

**DOI:** 10.1371/journal.pone.0093329

**Published:** 2014-03-25

**Authors:** Saniya Fayzullina, Lee J. Martin

**Affiliations:** Division of Neuropathology, Department of Pathology, and the Pathobiology Graduate Program, Johns Hopkins School of Medicine, Baltimore, Maryland, United States of America; University of Edinburgh, United Kingdom

## Abstract

Spinal Muscular Atrophy (SMA) is a hereditary childhood disease that causes paralysis by progressive degeneration of skeletal muscles and spinal motor neurons. SMA is associated with reduced levels of full-length Survival of Motor Neuron (SMN) protein, due to mutations in the *Survival of Motor Neuron 1* gene. The mechanisms by which lack of SMN causes SMA pathology are not known, making it very difficult to develop effective therapies. We investigated whether DNA damage is a perinatal pathological event in SMA, and whether DNA damage and cell death first occur in skeletal muscle or spinal cord of SMA mice. We used a mouse model of severe SMA to ascertain the extent of cell death and DNA damage throughout the body of prenatal and newborn mice. SMA mice at birth (postnatal day 0) exhibited internucleosomal fragmentation in genomic DNA from hindlimb skeletal muscle, but not in genomic DNA from spinal cord. SMA mice at postnatal day 5, compared with littermate controls, exhibited increased apoptotic cell death profiles in skeletal muscle, by hematoxylin and eosin, terminal deoxynucleotidyl transferase dUTP nick end labeling, and electron microscopy. SMA mice had no increased cell death, no loss of choline acetyl transferase (ChAT)-positive motor neurons, and no overt pathology in the ventral horn of the spinal cord. At embryonic days 13 and 15.5, SMA mice did not exhibit statistically significant increases in cell death profiles in spinal cord or skeletal muscle. Motor neuron numbers in the ventral horn, as identified by ChAT immunoreactivity, were comparable in SMA mice and control littermates at embryonic day 15.5 and postnatal day 5. These observations demonstrate that in SMA, disease in skeletal muscle emerges before pathology in spinal cord, including loss of motor neurons. Overall, this work identifies DNA damage and cell death in skeletal muscle as therapeutic targets for SMA.

## Introduction

Spinal Muscular Atrophy (SMA) is a genetic disorder characterized by progressive symmetrical limb and trunk paralysis, muscle atrophy, and motor neuron (MN) degeneration. It is caused by mutations in the *Survival of Motor Neuron* (*SMN1*) gene and is the second most common genetic cause of childhood mortality [1]. Based on severity of symptoms and time of onset, four childhood types of SMA have been identified. Type 0 SMA is the most severe, with prenatal onset characterized by reduced fetal movements, and joint contractures and muscle atrophy at birth [2]. Type I SMA has a clinical onset before the age of 6 months and death before the age of 2 years [3], [4]. Type II SMA patients live past 2 years of age and can sit but are never able to walk. Type III SMA patients experience onset of symptoms after 18 months of age, and are able to walk but have muscle weakness and decreased endurance [3], [5]–[9]. *SMN1* mutations result in reduced levels of full-length SMN protein. The *SMN2* gene, nearly homologous to *SMN1*, also produces SMN protein and thus modulates disease severity. However, a nucleotide change in *SMN2* results in post-transcriptional exclusion of Exon7 in most mRNA transcripts, resulting in a modified SMN2 protein [7], [10]–[13]. The amount of SMN dose compensation from *SMN2* depends on the number of copies of *SMN2*, which varies widely between individuals [14]–[16]. Currently, there are no therapies for SMA patients, because little is known about the function of SMN and the pathobiology of the disease, except that it affects MNs.

It remains unclear whether disease-initiating insults in SMA occur in neurons, skeletal muscle, or both tissues. This distinction is important because potential disease-modifying interventions may have greater therapeutic impact by preferentially targeting central nervous system versus peripheral tissues. It is also important because design and screening of SMA therapies is currently influenced by whether a candidate compound has blood-brain-barrier permeability. Muscle weakness and muscle loss are apparent early on in the disease, while there is a small loss of MNs [17]. Skeletal muscle innervation by MNs appears to be present in SMA patients, but neuromuscular junctions (NMJs) are not fully developed [18], [19]. While it is known that SMN is ubiquitously expressed throughout the body [20], there are no studies linking SMN tissue distribution to disease pathology. Likewise, although SMN is necessary for spliceosome assembly and pre-mRNA splicing [21], there is no conclusive evidence that aberrant splicing causes SMA disease. Pathology in SMA may be related to subcellular mislocalization, lack of function, or aberrant function of SMN in MNs and in other cell types [22]–[29]. A popular hypothesis is that MN disease leads to muscle denervation, ultimately resulting in muscle atrophy [24], [30]–[32]. Alternatively, MN disease may be a secondary result of skeletal muscle pathology that causes NMJ abnormalities [33], [34]. It is also possible that MNs and skeletal muscles undergo independent SMA-related injury [25], [34]–[37], further compounded by their interdependence via the NMJ [38]. MNs that undergo cell death cannot be endogenously replaced, but skeletal muscle has regenerative potential due to muscle-specific stem cells, called satellite cells [39]–[42]. If SMA is indeed a disease of skeletal muscle, this regenerative potential can be targeted for SMA therapies.

We studied cell death in a mouse model of SMA. Based on the broad clinical phenotypes implicating skeletal muscle involvement in SMA onset and severity, we hypothesized that disease-related cell death emerges in skeletal muscle before spinal cord. We used a mouse model of severe SMA [43] to ascertain the extent of cell death and DNA damage at different time points in embryonic and newborn mice. We show that cell death is evident first in skeletal muscle before spinal cord, and is more severe in skeletal muscle than in spinal cord. Moreover, abnormal DNA quality control is a possible molecular mechanism in SMA pathogenesis. These findings suggest that skeletal muscle degeneration and DNA repair may be relevant therapeutic targets in SMA.

## Materials and Methods

### Ethics Statement

This study was carried out in strict accordance with the recommendations in the *Guide for the Care and Use of Laboratory Animals* of the National Institutes of Health. The protocol (MO13M391) was approved by the Johns Hopkins University Animal Care and Use Committee. All efforts were made to minimize animal stress and suffering. Mice were housed no more than 5 per cage, with food and water ad libitum. Pups were handled as little as possible, and kept with dams until immediately prior to euthanasia. Mice were anesthetized and euthanized by decapitation or CO_2_ inhalation according to IACUC guidelines.

### Mouse Model of SMA

The “Taiwanese” SMA mouse model was used [43]. A breeding pair (stock 005058) was purchased from The Jackson Laboratory (Bar Harbor, ME) to establish a breeding colony. Genotyping was performed by PCR (mouse *Smn* and human *SMN2* transgene) and quantitative PCR (human *SMN2* copy number) according to protocols provided by the original investigators [43] on the Jackson Laboratory website (http://www.jax.org). Severe SMA mice were homozygous for the mouse *Smn exon 7* deletion and heterozygous for the human *SMN2* transgene. Control mice were heterozygous for the mouse *Smn exon 7* deletion and heterozygous for the human *SMN2* transgene. Matings were timed by the appearance of the vaginal plug (assumed to be embryonic day E0.5).

### Tissue Collection and Processing

Each embryo or pup was dissected separately; embryos were dissected in PBS on ice. Tissues were collected using a dissection microscope. The tail was collected for genotyping. For Southern blotting and gene array analysis, spinal cord, back muscles, and hindlimb muscles were immediately frozen on dry ice and stored at −80°C. For histology at P5, whole hindlimbs and spinal cords were immediately fixed in 4% paraformaldehyde for 3–4 hours, and then cryoprotected in 10% sucrose overnight and 30% sucrose for 6 hours. Hindlimbs were then frozen whole in OCT compound (Tissue-Tek). Spinal cords were cut into 4 sections (cervical, thoracic, lumbar, sacral) and frozen side-by-side in the same block of OCT compound. For histology at E15.5, embryos were fixed in 4% paraformaldehyde for 16 hours, and cryoptorected in sucrose as above. Whole embryos were then embedded in OCT compound. Frozen OCT-embedded tissues were sectioned transversely at 10 μm using a Leica cryostat. For histology at E13, embryos were fixed in 4% paraformaldehyde for 3–4 hours, followed by changes in graded ethanols, methyl salicylate, and cedarwood oil. Whole embryos were then embedded in paraffin. Paraffin-embedded tissues were sectioned transversely at 7 μm using a rotary microtome. Hematoxylin and eosin (H&E) staining was used for conventional pathological assessments and identification of anatomical landmarks.

### TUNEL Assay for Cell Death

The TUNEL assay detects DNA damage via terminal deoxynucleotidyl transferase (TdT)-mediated labeling of 3′ ends of double- and single-stranded DNA [45]. It is also a well-established assay for cell death [44], [45]. Transverse sections were thawed and dried for 1–3 hours, defatted and rehydrated, permeabilized with Cytonin reagent, and labeled using a fluorescence-based commercial kit (Trevigen Inc., Gaithersburg, MD). Sections were additionally labeled with Hoechst nuclear dye. Images were collected using a Zeiss fluorescent microscope with a 10x objective, using consistent exposure and gain settings. The red channel was used to capture autofluorescent signal (e.g. red blood cells) to exclude autofluorescent structures from TUNEL quantitation. Images were quantitated using SlideBook software’s mask functions: an intensity-gated mask for the fluorescent signal was combined with a manually-traced mask for each muscle group or spinal cord area. To ensure unbiased analysis, muscle group tracing was performed on the Hoechst and autofluorescence image, with the TUNEL channel turned off. Some muscles were not traced in their entirety because their borders could not be identified conclusively across all sections. All automatically selected pixels that colocalized with autofluorescent structures were manually excluded from analysis. For muscle, the total area of the TUNEL-positive muscle combined mask was normalized to the total area of the muscle mask, and used for statistical analysis. For spinal cord, the total number of automatically-defined TUNEL-positive objects was normalized to the total area of the ventral horn mask. Adjacent sections were stained with H&E to aid in anatomical identification of muscle groups and spinal cord areas in fluorescent images. For P5 spinal cord, 2–4 sections per mouse at each level were analyzed by TUNEL; values from these sections for each mouse were averaged before being used for graphs and statistical analysis. For E15.5 spinal cord and back muscle, 2 sections per mouse embryo at different lumbar levels were analyzed and 1 cervical section per mouse was analyzed. The spinal cord and adjacent back muscle were analyzed in the same sections.

### Pax7 and Laminin Immunofluorescent Analysis of Muscle

Tissue sections were prepared as described above for the TUNEL method. Fixed and rehydrated tissues were subjected to antigen retrieval in 0.01 M sodium citrate buffer with 0.05% Tween20 for 25 minutes at 95°C. After cooling, sections were blocked in 5% goat serum and 0.05% Tween20, followed by overnight incubation in primary antibody at 4°C. Sections were then incubated with Texas Red- conjugated goat-anti-mouse or goat-anti-rabbit secondary antibody for 2 hours at room temperature. The following primary antibodies were used: 1∶100 anti-Pax7 mouse monoclonal antibody (DSHB, Iowa City, Iowa) and 1∶200 anti-laminin rabbit antibody (L9393; Sigma, St. Louis, MO). Negative control sections had primary antibody omitted. After immunostaining, sections were stained by the TUNEL method. Nuclei were stained with a Hoechst dye. Confocal fluorescent images were captured on a Zeiss LSM510-Meta laser scanning microscope using a 100x objective. ZEN imaging software (Carl Zeiss International) was used to capture images, average pixels (3 pixels in x and y dimensions), and adjust channel histograms. Conventional fluorescent images were captured and edited using a 40x objective, as described above.

### ChAT and TUNEL Immunofluorescent Analysis of Spinal Cord

Choline acetyl transferase (ChAT) immunofluorescence and the TUNEL staining method were combined in a separate set of P5 tissue sections and E15.5 tissue sections. Frozen tissue sections prepared as described above were allowed to thaw and dry at room temperature for 1–2 days, followed by rehydration in PBS for 1 hour. Sections were permeabilized with 0.5% Tween20, 0.05% Triton X100 in PBS for 1 hour, and blocked in 10% donkey serum and 0.05% Tween20 for 1 hour, all at room temperature. Sections were then incubated overnight in goat anti-ChAT primary antibody (AB144P; Millipore) at 4°C. Following washes, sections were incubated with AF594-conjugated donkey-anti-goat secondary antibody (A11-058; Life Technologies) for 2 hours at room temperature. After immunostaining, sections were stained by the TUNEL method as described above. Nuclei were stained with a Hoechst dye. Conventional fluorescent images were captured using a 10x objective and edited as described above. For TUNEL quantitation, images were processed and analyzed as above. For ChAT quantitation, cell nuclei surrounded by ChAT-positive cytoplasm were counted within the ventral horn of the spinal cord. ChAT counts were normalized to the total area of the traced ventral horn. There were very few TUNEL-positive puncta in E15.5 ventral horn and back muscle. Because E15.5 sections included the entire body of the embryo, we were able to validate that the TUNEL method worked on these sections by detecting numerous TUNEL-positive nuclei in the liver and skin.

### Southern Blotting

Southern blotting was used as a biochemical method to complement the histology-based TUNEL assay. DNA was extracted from frozen tissues and treated with RNase. TdT was used to label extracted DNA with digoxigenin (DIG)-dUTP fragments. Labeled DNA was separated on a 1.3% agarose gel with a DIG-conjugated molecular weight ladder (MW Marker VI, Roche Applied Science) and transferred to a nylon membrane. The digoxigenin-labeled DNA was visualized using a commercial chemiluminescence-based kit (Roche Applied Science). X-ray film was exposed to the chemiluminescent signal and scanned to produce a digital copy. Blots were analyzed using ImageJ software (NIH; Bethesda, MD). The integrated density of the top band of each lane was divided by the integrated density of the bottom band. This measurement was intended to show whether the DNA was predominantly in the top of the lane (no DNA damage) or in the bottom portion of the ladder (DNA damage).

### Gene Expression Array

RNA was extracted using Trizol reagent (Invitrogen) from frozen mouse back muscles collected at P2, and cDNA was synthesized using the RT^2^ First Strand kit (Qiagen). RNA quality was assessed by microarray on the Agilent 2100 Bioanalyzer. All RNA samples had RNA integrity numbers (RIN) between 9 and 10. Gene array plates were purchased from Qiagen (SABiosciences array PAMM-029ZD-12, “DNA Damage Signaling Pathway”). Biorad CFX-96 bioanalyzer was used to run quantitative PCR reactions with RT^2^ SYBR Green qPCR mastermix (Qiagen). All kits were used according to manufacturer instructions. Arrays included positive reverse transcription controls, positive qPCR controls, and genomic DNA contamination controls. All control results were valid within manufacturer specifications. Back muscle RNA samples from 3 mice (3 biological replicates) were assayed per group. Raw ΔCt values for all genes of interest were normalized to *Gapdh* ΔCt. Other housekeeping genes were included in the array, but Gapdh resulted in the most consistent expression levels between mice and between groups, and Gapdh protein expression had been previously validated on samples from the same mice by Western blot. Array results were analyzed using an SABiosciences custom Microsoft Excel spreadsheet to calculate fold change (SMA average (2∧-ΔCt)/control average (2∧-ΔCt)) and *p* values. The raw data from this microarray are publicly available through NCBI’s Gene Expression Omnibus (GEO), series accession number GSE55481 (http://www.ncbi.nlm.nih.gov/geo/query/acc.cgi?acc=GSE55481).

### Electron Microscopy (EM) Analysis of Muscle

Mice at P6 were euthanized by CO_2_ inhalation, quickly perfused through cardiac puncture with ice cold HEPES buffer (0.1 M HEPES, 1% sucrose, 3 mM CaCl_2_, pH 7.2) to exsanguinate, followed by perfusion with ice cold 2% paraformaldehyde and 2% glutaraldehyde in HEPES buffer. Whole legs were post-fixed in the same paraformaldehyde/glutaraldehyde mixture overnight at 4°C. Muscle groups were dissected out under a dissection microscope and placed in HEPES buffer. Tissues were then fixed in osmium tetroxide, followed by dehydration in alcohols, changes in propylene oxide and resin, and embedding in resin. All tissue pieces were oriented in resin blocks using a dissection microscope to ensure the same orientation between mice. Thin (100 nm) sections were cut onto copper grids and stained with uranyl acetate and lead citrate. Sections were imaged digitally using a Hitachi transmission electron microscope.

### Myofiber Measurement

Mice at P4 were euthanized and tissues processed as described for EM analysis above. Semi-thin (1 μm) plastic sections of tibialis anterior (TA) and rectus femoris (RF) muscles were cut onto glass slides and stained with toluidine blue. These preparations can be imaged at high-resolution using light microscopy. Sections from different mice were at similar anatomical levels. Sections were imaged using a conventional brightfield microscope with a digital camera. Multiple images were collated to cover the entire area of the muscle. Measurements of myofiber area and number of cells per area were made using ImageJ software (NIH; Bethesda, MD). A digital grid was placed over the image, and quantitation was made in squares of the grid along diagonals across the long axis of the muscle. The aim of this method was to cover as much of the muscle as possible using non-adjacent, semi-random samples. For myofiber number counts, grid squares with major anatomical structures, such as large blood vessels and nerves were avoided, so as not to bias the counts. Myofiber counts were normalized to the total area (in pixels) of the grid squares counted.

### Western Blotting

Whole spinal cords and leg muscles (multiple muscle groups from lower and upper leg combined) were homogenized with protease inhibitors (Complete Protease Inhibitor Cocktail; Roche). Protein homogenates were electrophoresed on Bis-Tris 4–12% gels (Biorad) or polyacrylamide gel (for XRCC1 only) with a protein ladder (Benchmark ladder; Life Technologies). After transfer to a nitrocellulose membrane, blots were stained with Ponceau S and digitally scanned. Ponceau staining was used to normalize data for loading differences. Blots were blocked in 2.5% milk and 0.1% Tween20 in TBS, followed by primary antibody (diluted in milk blocker) incubation overnight at 4°C, and HRP-conjugated secondary antibody in milk blocker for 2 hours at room temperature. Immunostaining was visualized using an ECL substrate and exposed to photographic film. Films were digitally scanned for analysis. The following primary antibodies were used: XRCC1 rabbit monoclonal (3631-1; Epitomics) diluted 1∶2000, phospho(Ser139)-H2AX mouse monoclonal (05-636; Millipore) diluted 1∶1000, phospho(S15/S18)-p53 rabbit polyclonal (AF-1043; R&D Systems) diluted 1∶1000, and phospho-RPA32 rabbit polyclonal (A300-246A; Bethyl) diluted 1∶4000. The following secondary antibodies were used: goat-anti-mouse-HRP and goat-anti-rabbit-HRP (various from Biorad).

### Statistical Analyses of TUNEL, ChAT, and Myofiber Measurement Data

All comparisons were made between littermates. There were n = 4 or 5 mice per group for all muscle and spinal cord comparisons at P5 (TUNEL and ChAT), n = 3 mice per group for myofiber measurements at P4, n = 2 (SMA) and n = 4 (control) mice per group at E15.5 (TUNEL and ChAT), and n = 3 mice per group at E13 (TUNEL). SMA and control groups were compared using a one-tailed unpaired t-test; each muscle group and each spinal cord level were compared separately. A Welch’s correction was used for groups where variances differed significantly. The *p* value for statistical significance was set to *p*<0.05 for all tests.

## Results

### Skeletal Muscle of SMA Mice Shows Numerous Apoptotic Profiles Shortly after Birth, but Myofiber Size and Number are Similar to Control

**Figure 1:**
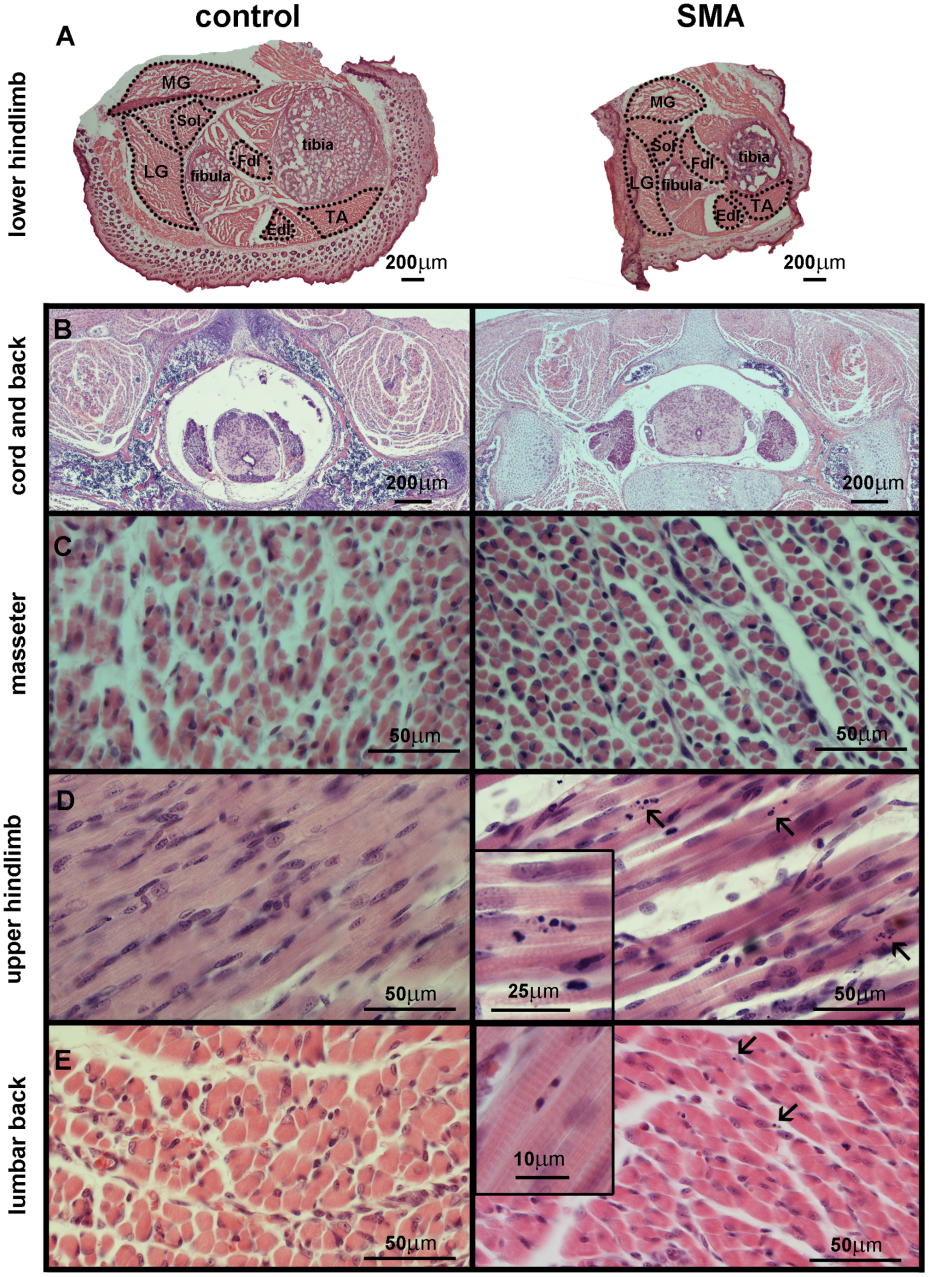
Skeletal muscle atrophy and apoptosis in SMA mice. H&E staining was performed on transverse sections of the lower hindlimb at P5 (**A**), and whole body at P4 (**B** – lumbar spinal cord and back muscles, **C** – masseter, **D** – forelimb muscles, **E** – lumbar back muscles). Representative skeletal muscle images from control (left column) and SMA mice (right column) are shown at the same scale. Arrows and insets indicate apoptotic profiles (condensed, fragmented nuclei). Dotted lines in **A** delineate areas quantified. Some muscle groups were not quantified in their entirety because they were not intact or were not identifiable on all sections. TA – tibialis anterior muscle, Fdl/Edl – flexor/extensor digitorum longus muscle, Sol – soleus muscle, LG – gastrocnemius lateralis muscle, MG – gastrocnemius medialis muscle.

We conducted a whole-body histological survey of SMA mice at postnatal days 4 and 5 (P4–P5) to identify muscle groups most affected by the disease. Although SMA mice were smaller than littermate controls, all muscle groups, spinal cord, and organ systems appeared to be anatomically normal (Figure 1A and 1B, data not shown). SMA skeletal muscles in the limbs and back contained numerous nuclear apoptotic profiles (Figure 1D and 1E). The masseter muscle did not exhibit apoptotic profiles in SMA mice (Figure 1C). Occasional apoptotic profiles were observed in littermate-matched control skeletal muscles. In affected muscles of SMA mice, apoptotic profiles were scattered throughout the muscle rather than being clustered. There was no obvious indication of major inflammatory changes or myofiber necrosis.

**Figure 2:**
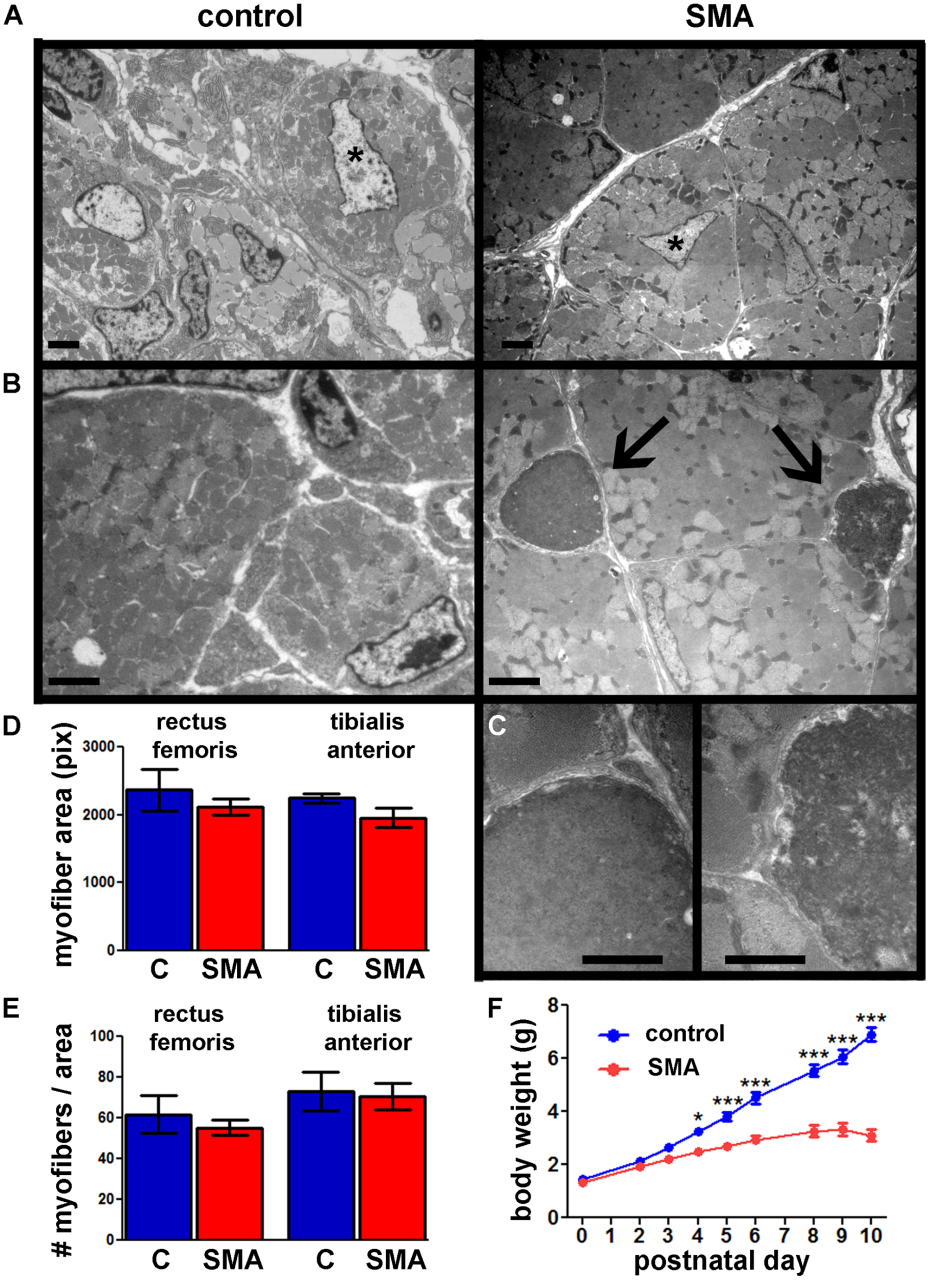
SMA mouse skeletal muscles exhibit apoptotic cell death in satellite cells, but normal myofiber size. **A, B, C**– transmission electron microscopy images of TA muscles from SMA mice (right) and control littermates (left) at P6. **A**. Central nuclei (asterisk) are infrequent, but can be seen in both control and SMA muscles. Scale bar = 2 μm. **B**. Apoptotic satellite cell nuclei (arrows) were observed in SMA muscles, but not control muscles. Adjacent myofibers appear normal. Scale bar = 2 μm. **C**. Magnified images of the two apoptotic cells shown in the panel above. Cell membranes are distinct from the adjacent myofiber membranes, indicating that these dying cells are satellite cells. The apoptotic profile shown at right is at a more advanced stage of apoptosis compared to the cell at left. Nuclear condensation, membrane involution, and membrane fragmentation can be seen. Scale bar = 1 μm. **D, E**. Myofiber area (**D**) and myofiber number per muscle area (**E**) measurements in TA and RF muscles from SMA mice (red) and control littermates (blue) at P5 (Mean ± SE; n = 3). C = control. There were no statistically significant differences (t-test). **F**. Body weights of SMA mice and control littermates (Mean ± SE; n = 4–5). Statistical significance between SMA and control (two-way repeated measures ANOVA, Bonferroni test): * *p*<0.05, ** *p*<0.01, *** *p*<0.001.

To confirm that myonuclei in SMA mice die by apoptosis, we analyzed TA muscles from P6 SMA mice and control littermates using electron microscopy (Figure 2). We observed multiple apoptotic profiles in SMA muscles, characterized by nuclear DNA condensation, cell shrinkage, and membrane involution and fragmentation (Figure 2B, 2C) [46]–[48]. We did not observe apoptotic profiles in the control muscle. We observed apoptosis in satellite cell nuclei (Figure 2C), indicating that this cell type is affected in SMA mice. We could not determine the extent of myofiber regeneration in SMA because we studied muscles in early postnatal development, which looks similar to regeneration. For example, both SMA and control muscles had a few centrally placed myofiber nuclei (Figure 2A), commonly used to identify regenerating fibers in adult muscle [49]. The number of central nuclei was too small to quantify dependably, but there were no apparent differences between SMA and control. We also did not observe extensive fibrosis or disorganized filaments within myofibers.

We then asked whether increased apoptosis in SMA mouse skeletal muscle causes a developmental defect resulting in smaller or fewer myofibers. Myofiber areas and myofiber numbers in TA and RF were not different between SMA and control mice at P5 (Figure 2D, 2E). At the same time, SMA mice have significantly smaller body weights (Figure 2F) and total muscle size (Figure 1A) at P5, accompanied by progressive muscle weakness (Videos S1–S7 in File S1). Together these data indicate that SMA mouse skeletal muscles retain myofibers that are normal in size and density, but there are fewer myofibers in the entire muscle, causing SMA mice to be smaller than littermate controls.

### Skeletal Muscles Exhibit Cell Death at P5 in SMA Mice, as Confirmed by TUNEL

**Figure 3:**
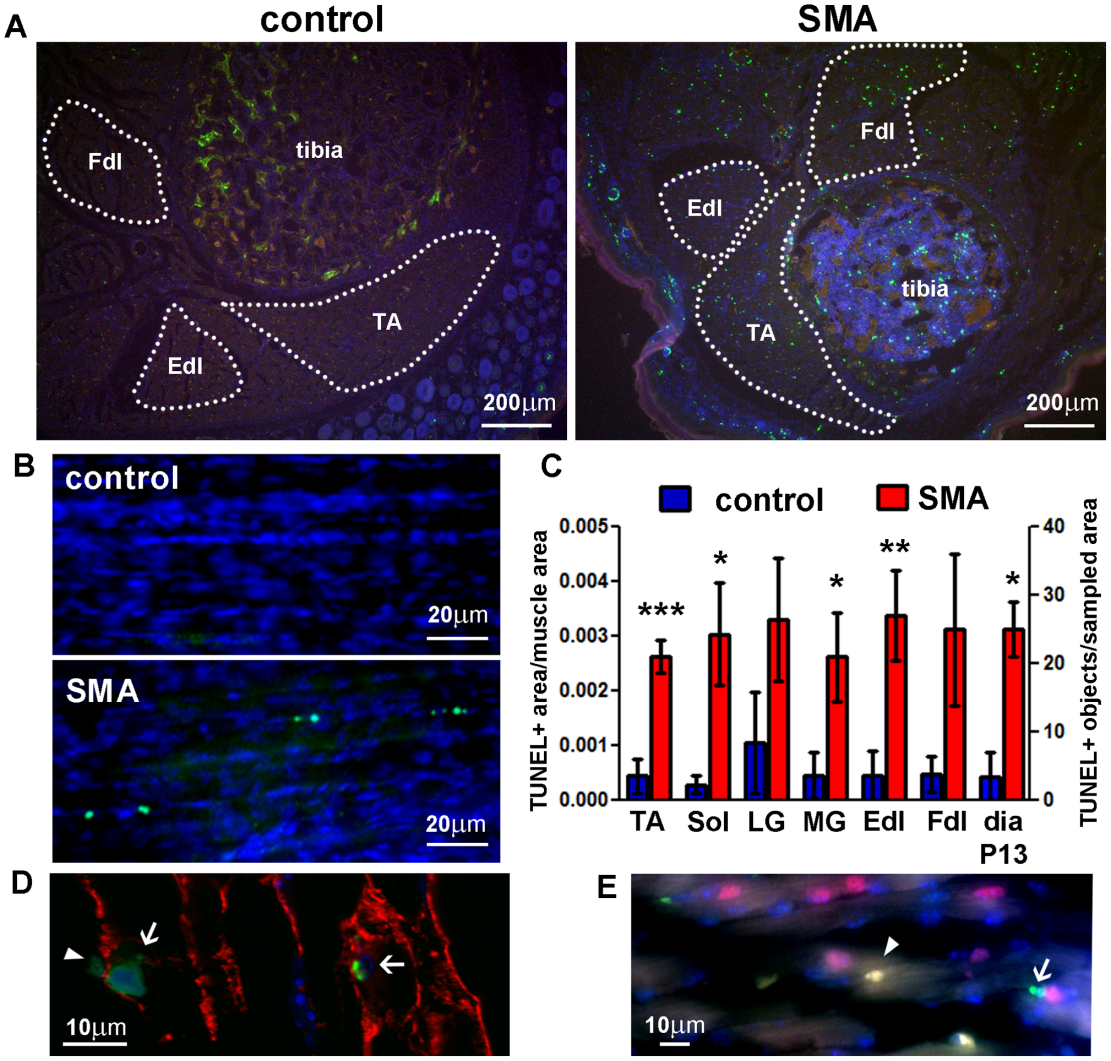
Cell death in SMA mouse skeletal muscle at postnatal days 5 and 13. **A, B**. TUNEL (green) was performed on transverse sections of the lower hindlimb at P5 (**A**) and whole diaphragms at P13 (**B**). Hoechst (blue) was used to stain cell nuclei. The red channel was used to exclude autofluorescent cells (e.g. red blood cells) from analysis. Dotted lines delineate the muscle areas quantified. **C**. TUNEL-positive counts in lower hindlimb skeletal muscles and diaphragm (Mean ± SE; n = 5–6 for hindlimb, n = 2 for diaphragm). TA – tibialis anterior muscle, Fdl/Edl – flexor/extensor digitorum longus muscle, Sol – soleus muscle, LG – gastrocnemius lateralis muscle, MG – gastrocnemius medialis muscle, dia – diaphragm. Statistical significance between SMA and control for each muscle group (one-tailed t-test): * *p*<0.05, ** *p*<0.01, *** *p*<0.001. Micrographs of additional hindlimb muscle groups are shown in Figure S1. TUNEL-positive objects per muscle area are shown for diaphragm; TUNEL-positive area per muscle area is shown for all other muscles. **D**. Confocal immunofluorescent image of hindlimb skeletal muscle at P5; green – TUNEL, red – laminin (myofiber cell membrane marker), blue – nuclei. **E**. Conventional immunofluorescent image of hindlimb skeletal muscle at P5; green – TUNEL, red – Pax7 (satellite cell marker), blue – nuclei. Arrows – myotube nuclei, arrowheads – satellite cell nuclei.

We used the TUNEL method to confirm the presence of cell death in SMA mouse skeletal muscle and to assay for DNA damage at a cell-specific resolution. Lower limb skeletal muscles of SMA mice at P5 exhibited increased numbers of TUNEL-positive nuclei, compared with littermate controls (Figure 3, Figure S1). We observed TUNEL-positive nuclei in myotubes and satellite cells (Figure 3D and 3E), although future studies will be required to determine if there is a cell type-specific vulnerability. In addition to limb weakness and overt skeletal muscle atrophy, defined here as a gross decrease in muscle mass (Figure 1A), SMA mice suffer from respiratory distress at endstage. To determine whether diaphragm degeneration accompanies this endstage phenotype, we analyzed the diaphragm using TUNEL at endstage disease (P13). As with other skeletal muscles, SMA mouse diaphragms exhibited an increase in TUNEL-positive nuclei, compared with age-matched littermate controls (Figure 3B and 3C).

### DNA Damage Begins in SMA Mouse Skeletal Muscle Prenatally before Spinal Cord DNA Damage

**Figure 4:**
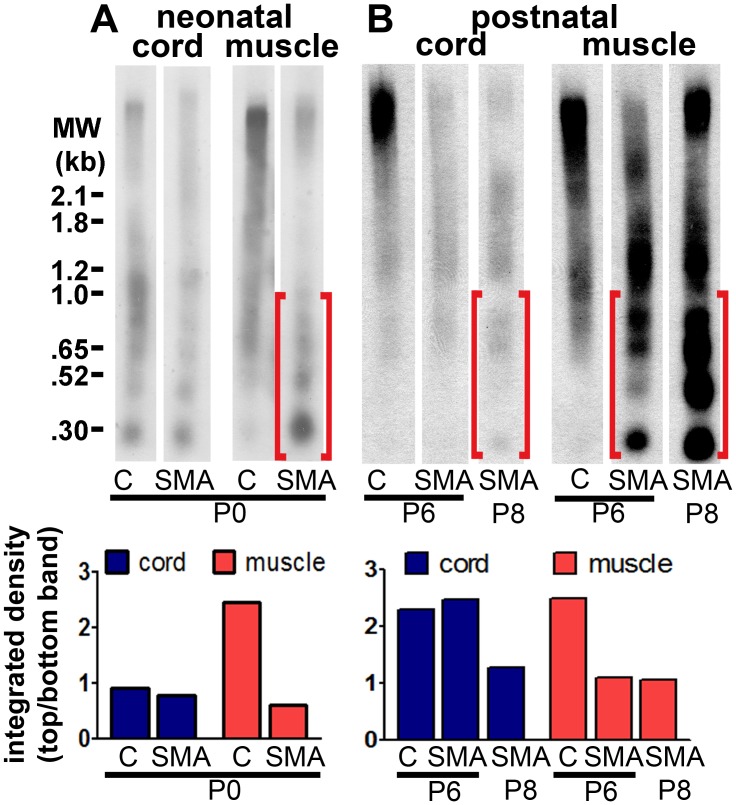
Internucleosomal fragmentation of DNA emerges in skeletal muscle before spinal cord in SMA mice. Whole genomic DNA from spinal cord and hind limb muscle (postnatal days P0–P8) was separated by gel electrophoresis. DNA breaks were end-labeled with DIG-dUTP by TdT and detected using a DIG-based Southern blot. A DIG-conjugated molecular weight ladder was run on the same gel (size marked on the left). Brackets show areas of lower molecular weight DNA in skeletal muscle, indicating DNA fragmentation. Substantial differences in internucleosomal fragmentation of DNA were not observed between control and SMA spinal cord until postnatal day 8. Bar graphs under each blot represent integrated density measurements of the top band in each lane divided by the bottom band in each lane. **A**. Southern blot of spinal cord and skeletal muscle DNA from SMA and control (“C”) littermates at postnatal day 0. **B**. Southern blot of spinal cord and skeletal muscle DNA from SMA and control (“C”) littermates at postnatal day 6 and a separate SMA mouse at postnatal day 8.

To confirm histological evidence for apoptosis observed in the H&E, TUNEL, and EM preparations, we assessed genomic DNA fragmentation using Southern blotting. We compared SMA mouse skeletal muscle and spinal cord at neonatal and postnatal time points to identify when cell death and DNA damage emerge during development. SMA mice at birth (P0) exhibited internucleosomal fragmentation in genomic DNA from hindlimb skeletal muscle (Figure 4A). SMA and control mice exhibited low levels of normal developmental cell death in the spinal cord at P0 (Figure 4A). At P6, SMA mice exhibited an increase in DNA fragmentation in muscle but not in spinal cord (Figure 4B). SMA mice exhibited increased DNA fragmentation in the spinal cord at P8, by which time SMA muscle had a very high level of DNA fragmentation (Figure 4B). Thus, this biochemical whole-tissue assessment suggested that DNA damage began in skeletal muscle prenatally, before spinal cord was affected.

### Spinal Cord Ventral Horn Motor Neurons do not Exhibit Cell Death at P5 in SMA Mice

SMA affects MNs in the ventral horn of the spinal cord in humans [3], [17]. MNs are a minority compared to other cellular constituents of the spinal cord. Thus, the DNA damage signal from MNs may be diluted to undetectable levels in whole tissue homogenates. To examine cell death at the cellular level, we assayed spinal cord sections from SMA mice by H&E and TUNEL.

**Figure 5:**
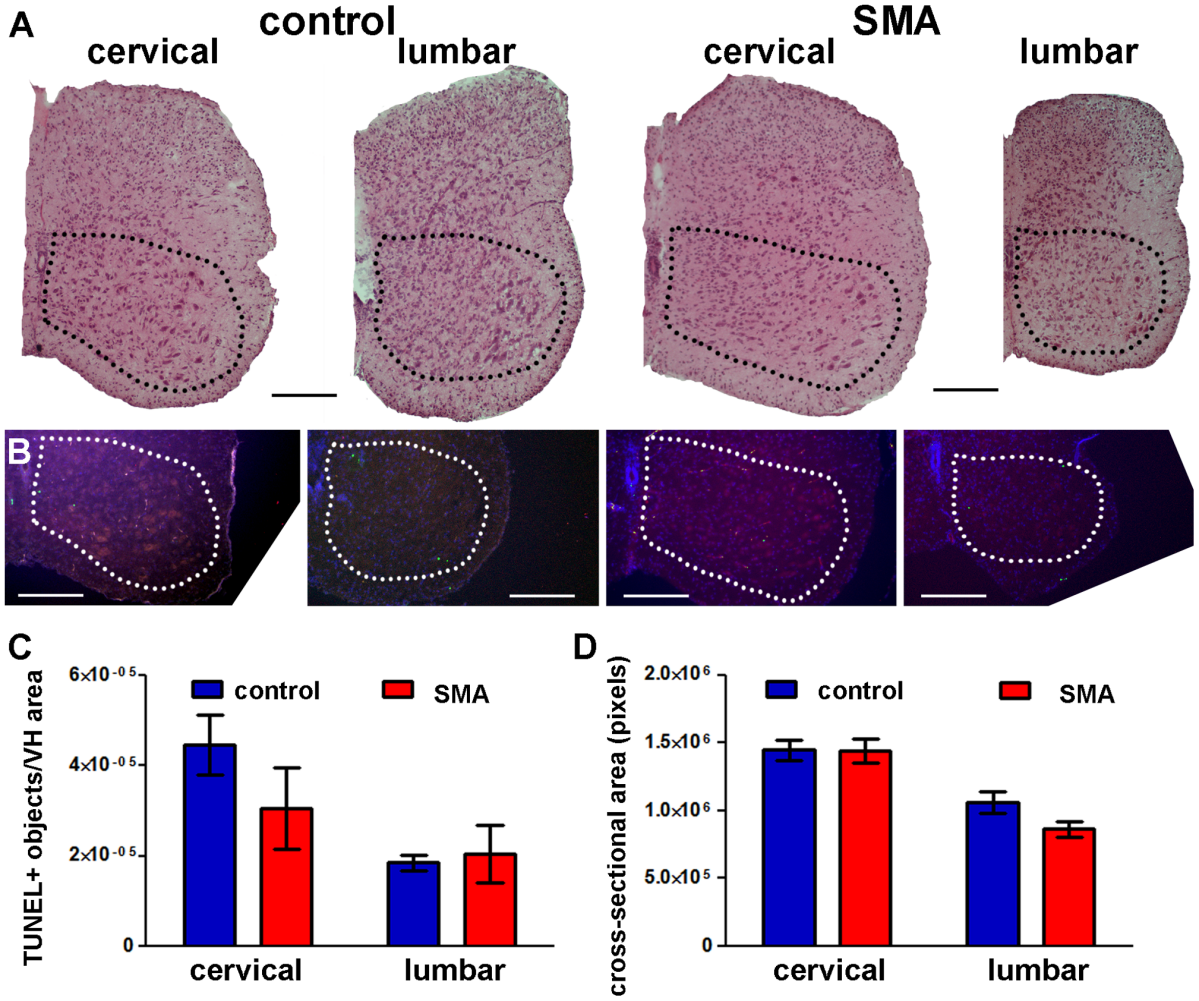
Cell death in P5 SMA mouse spinal cord is not increased significantly. **A**. Representative images of H&E-stained transverse sections at P5 showing cervical and lumbar levels of spinal cord from control (left) and SMA (right) littermates. **B**. TUNEL (green) was performed on transverse whole body sections at P5. Hoechst (blue) was used to stain cell nuclei. A separate channel (red) was used to identify anatomical landmarks and exclude autofluorescent signal (e.g. red blood cells). **A** and **B**: dotted lines delineate the ventral horn (VH) areas analyzed; all scale bars = 200 μm. **C**. TUNEL-positive counts in the VH of spinal cord at lumbar and cervical levels (Mean ± SE; n = 4–6). **D**. Cross-sectional area of spinal cord at lumbar and cervical levels (Mean ± SE; n = 3–6). There were no statistically significant differences between SMA and control groups (one-tailed t-test).

**Figure 6:**
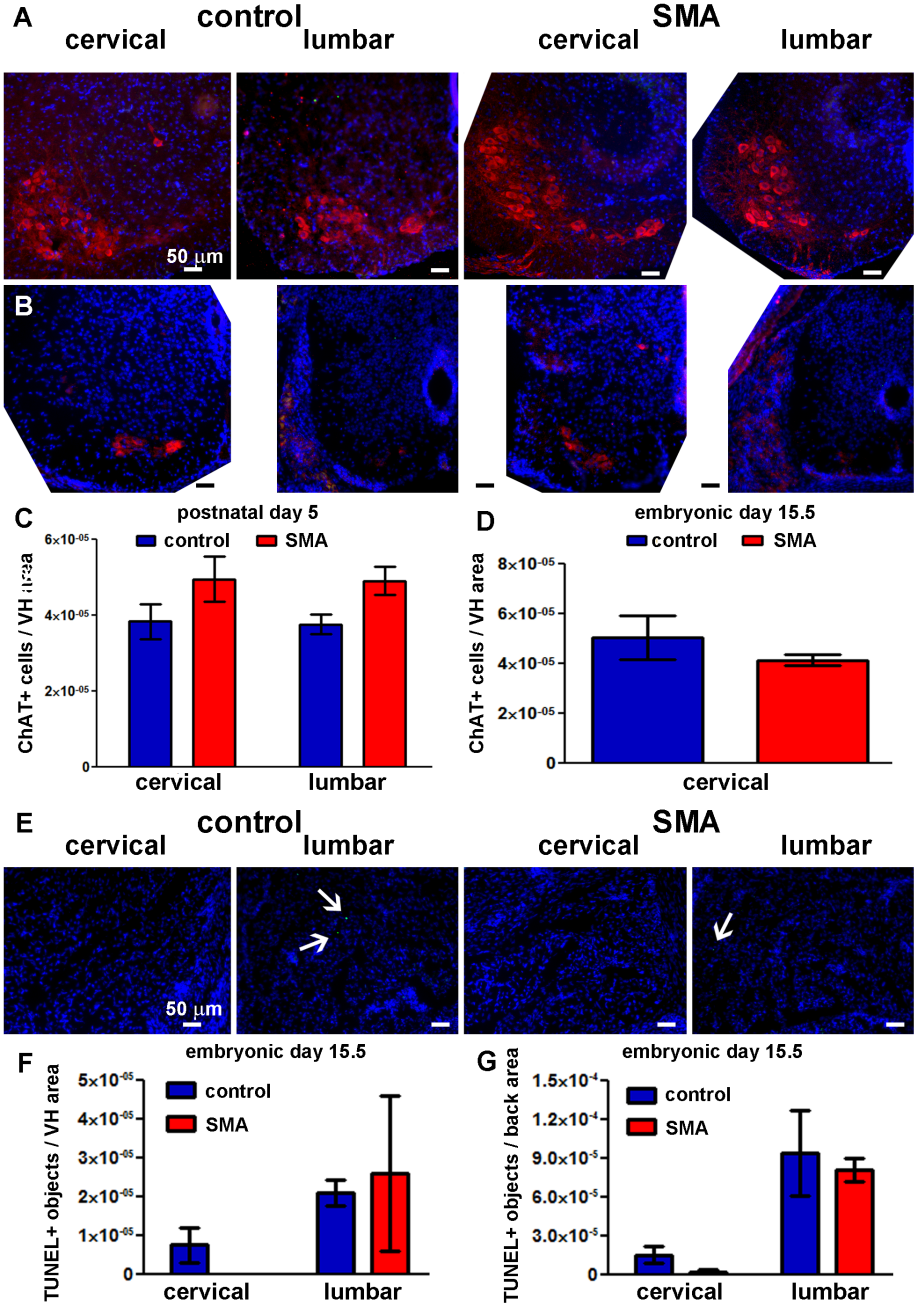
No significant motor neuron loss in spinal cord at E15.5 or P5 in SMA mice. **A, B, C**. TUNEL (green), ChAT immunostaining (red), and nuclear staining (blue) were performed on transverse whole body sections at E15.5 (**B**) and isolated spinal cord sections at P5 (**A**). Representative images of lumbar and cervical spinal cord are shown for the control (left) and SMA (right) mouse groups. Scale bar = 50 μm in all panels. **C, D**. ChAT-positive motor neuron counts in the VH of spinal cord at lumbar and cervical levels at P5 (**C**; Mean ± SE; n = 4–5) and cervical level at E15.5 (**D**; Mean ± SD; n = 2 (SMA), n = 4 (control)). **E**. TUNEL (green) and nuclear staining (blue) were performed on transverse whole body sections at E15.5. Representative images of lumbar and cervical ES back muscles are shown for the control (left) and SMA (right) littermates. Scale bar = 50 μm in all panels. Arrows denote TUNEL-positive nuclei. **F, G**. TUNEL-positive structure counts in spinal cord VH (**F**) and ES muscle (**G**) at lumbar and cervical levels at E15.5 (Mean ± SD; n = 2 (SMA), n = 4 (control)). There were no statistically significant differences between SMA and control groups (two-tailed t-test).

The same affected SMA mice that exhibited many TUNEL-positive cells in skeletal muscle exhibited no overt pathology in large cell bodies in the ventral horn, presumed to be MNs (Figure 5A) and no increase in TUNEL-positive cells in the ventral horn of the lumbar and cervical spinal cord (Figure 5B and 5C). Co-staining with an antibody against the MN marker ChAT combined with TUNEL confirmed that there were no TUNEL-positive MNs in P5 spinal cords of SMA and control mice (Figure 6A). Counts of ChAT-positive MNs also confirmed that there were no differences in MN numbers between SMA and control littermates (Figure 6C). Although SMA mice were significantly smaller than control littermates (Figure 2F), there were no statistically significant differences in spinal cord cross-sectional area between groups (Figure 5D).

### The Accumulation of DNA Damage in SMA Mouse Skeletal Muscle Begins after Embryonic Day 15.5

**Figure 7:**
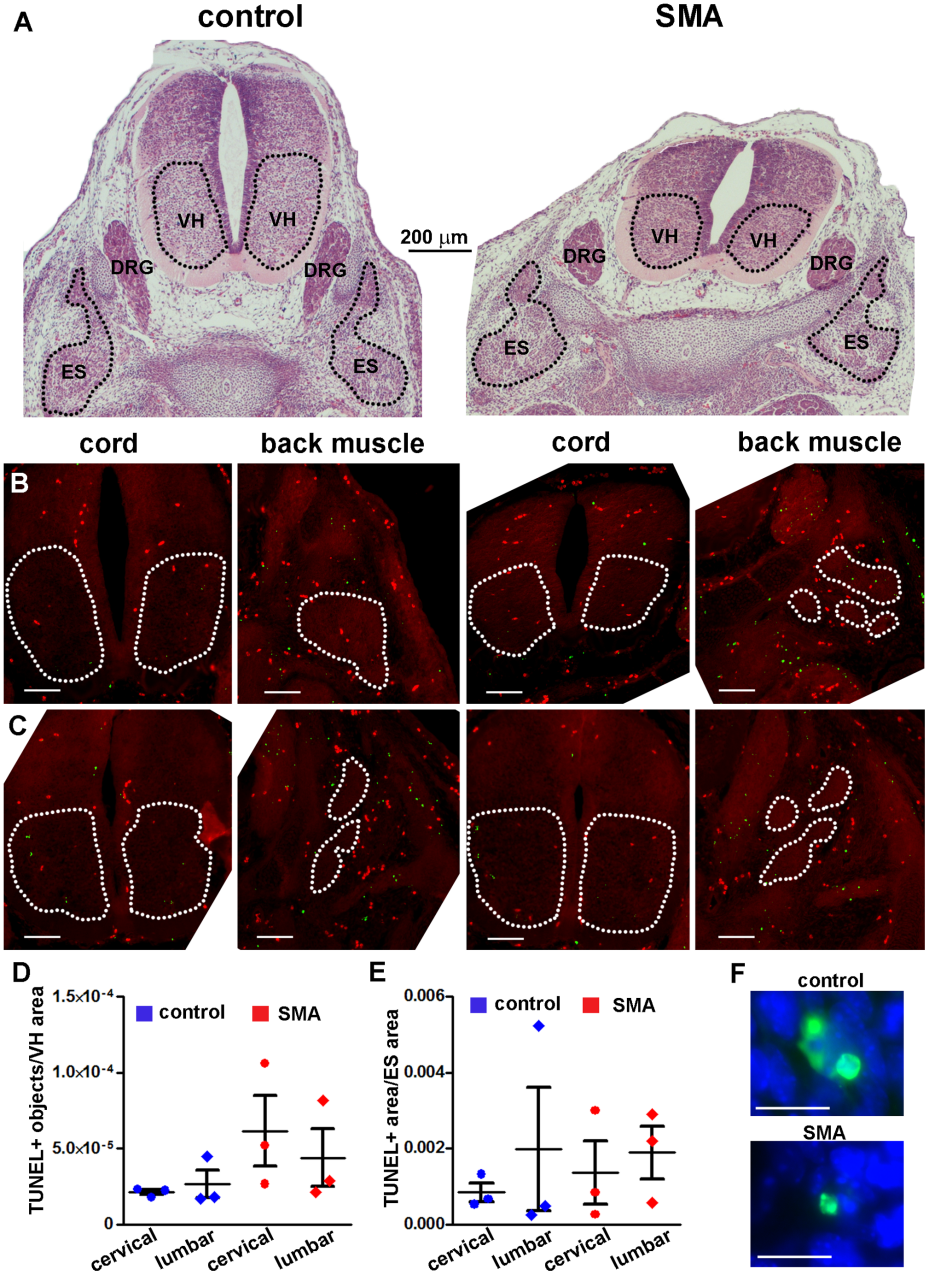
Cell death in E13 SMA mouse skeletal muscle or spinal cord is not increased significantly. **A**. Representative images of H&E-stained transverse sections of the whole body at E13 showing lumbar levels of spinal cord back from control (left) and SMA (right) littermates. Images are shown at the same scale. The areas delineated as ventral horn (VH) of spinal cord and erector spinae muscle group (ES) were analyzed. **B, C**. TUNEL (green) was performed on transverse whole body sections at E13. A separate channel (red) was used to identify anatomical landmarks and exclude autofluorescent signal (e.g. red blood cells). The total red signal was subtracted from the total green signal to show TUNEL-specific signal only. Representative images of lumbar (**B**) and cervical (**C**) spinal cord and back muscles are shown for the control (left) and SMA (right) mouse groups. Scale bar = 100 μm. **D**. TUNEL-positive structure counts in the VH of spinal cord at lumbar and cervical levels (individual counts and Mean ± SE; n = 3). **E**. TUNEL-positive structure counts in ES muscle at lumbar and cervical levels (individual counts and Mean ± SE; n = 3). There were no statistically significant differences between SMA and control groups (two-tailed t-test). **F**. Representative high-magnification images identifying TUNEL-positive structures (green) in E13 control and SMA mouse skeletal muscle as apoptotic nuclei. Hoechst (blue) was used to stain cell nuclei. Scale bar = 10 μm.

We analyzed cell death in skeletal muscle and spinal cord at embryonic time points, E13 and E15.5, using TUNEL (Figure 6, Figure 7). Spinal cord ventral horns and adjacent erector spinae (ES) back muscles at cervical and lumbar levels were analyzed (Figure 6B and 6E, Figure 7B and 7C). SMA mice at E13 had higher mean counts of TUNEL-positive nuclei in spinal cord ventral horn, compared with littermate controls (Figure 7D). However, the increases in TUNEL staining were not statistically significant. E13 erector spinae (ES) muscles also showed a trend for increased TUNEL-positive nuclei at cervical levels, but ES muscles at lumbar levels did not (Figure 7E). At E15.5, there were very few TUNEL-positive cells in both SMA and control ventral horn and ES muscles, and no statistically significant differences at the lumbar or cervical levels (Figure 6F and 6G). Therefore, histological analyses showed that DNA damage in SMA begins after the E15.5 embryonic time point. Furthermore, at E15.5 there were no differences in the numbers of ChAT-positive MNs in the cervical spinal cord between SMA and control mice (Figure 6B and 6D). These data indicate that there is no global loss of ventral horn MNs in SMA mice at early- to mid-embryogenesis.

### SMA Mouse Skeletal Muscle Pathology is not Accompanied by Changes in Expression of DNA Damage Response and Repair Genes

**Figure 8:**
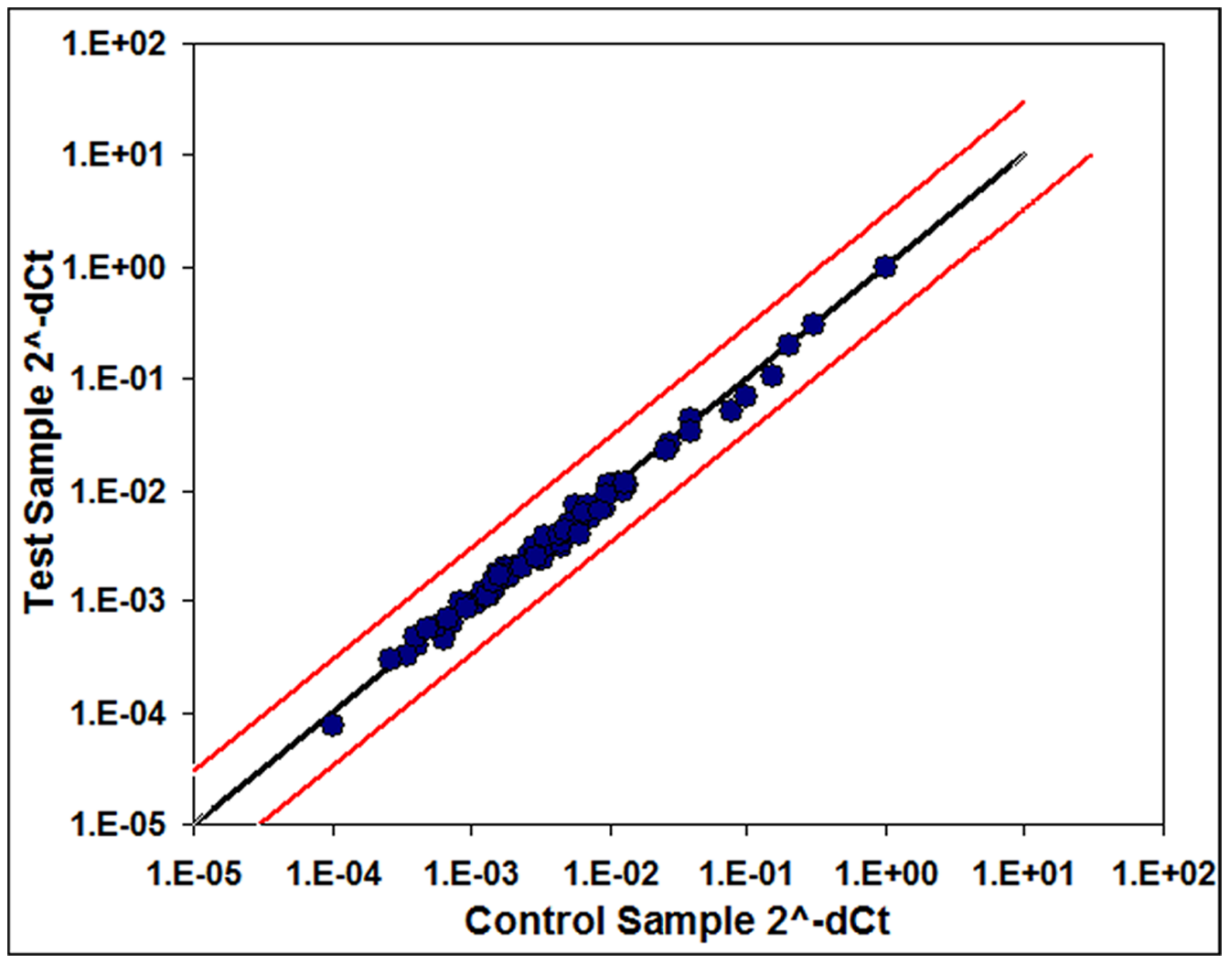
Expression of DNA damage signaling genes in P2 SMA skeletal muscle is not increased significantly. A commercially available gene array was used to assay skeletal (back) muscle from SMA mice and control littermates (n = 3). There were no statistically significant changes in the expression of 84 genes (see Table S1 for a complete list of genes). Each circle represents a single gene assayed. The black line denotes a fold-change of 1. Red lines denote a fold-change of 3.

Because of the extensive postnatal skeletal muscle DNA damage and cell death (Figures 1–3), we assessed skeletal muscle for activation of cell death and DNA damage pathways by microarray. The morphology of damaged nuclei identified by H&E and TUNEL indicated DNA condensation and fragmentation associated with apoptosis (Figure 7F) [50]. We therefore profiled P2 SMA muscle for DNA damage signaling and apoptosis gene expression by RNA microarray. Back skeletal muscles from SMA mice and control littermates were used. No changes were detected in any of the 84 genes on the array (Table S1, Figure 8). To supplement the negative gene array results, we performed Western blot analysis for several proteins involved in response to DNA damage and DNA repair: phospho-RPA32 [51], phospho-p53 [52], XRCC1 [53], and phospho(Ser139)-H2AX [54]. RPA is a multi-subunit protein complex necessary for DNA repair by multiple mechanisms. Its 32kD subunit (encoded by the *Rpa2* gene) is phosphorylated in response to DNA damage [51]. Although the *Rpa1* gene (encoding the 70kD subunit) was included in the array, *Rpa2* was not. XRCC1 is necessary for repair of single-strand DNA breaks [53], and was included in the gene array. Although *p53* and *H2ax* were included in the gene array, the array would not detect phosphorylation of their protein products that is the hallmark of DNA damage and cell death pathway activation. Western blot results agreed with gene array results: there were no consistent differences between XRCC1, phospho-p53, phospho-H2AX, and phospho-RPA32 protein levels in SMA and control muscle and spinal cord (Figure S2).

## Discussion

We have identified an early vulnerability of skeletal muscle that distinguishes it from spinal cord in SMA mice. We observed DNA fragmentation in the skeletal muscles of SMA mice as early as P0 by Southern blot. We did not find significant cell death in the spinal cord ventral horn as late as P5, and DNA fragmentation was not detected in spinal cord until P8. These observations suggest that in SMA, disease in skeletal muscle emerges before cell death in spinal cord.

Historically, SMA research has focused on MN disease as the primary mechanism and treatment target, with muscle pathology assumed to be secondary to the degeneration of MNs [24], [30], [31], [55]. However, recent studies in mouse models of SMA have presented evidence for both MN-driven and skeletal muscle-driven etiology of the disease. Selective deletion of mouse *Smn* in either skeletal muscle [56] or spinal cord MNs [57], [58] leads to SMA-like symptoms. However, MN-specific depletion of SMN leads to milder symptoms compared with SMN depletion in all tissues [58]. Gavrilina et al. used the severe “Burghes” mouse model of SMA to show that selective expression of SMN in the central nervous system (using the prion promoter) rescues this model, but selective expression in skeletal muscle (using the skeletal muscle actin promoter) does not affect disease progression [59]. Caveats of this work include driver strength of the promoter used and tissue specificity of the promoter expression. The prion promoter is not exclusively nervous system-specific and can drive some expression of SMN in skeletal muscle [59]. A group working with the “Taiwanese” model of SMA (the model used here) showed that peripheral induction of full-length SMN expression is necessary for complete rescue of this model [60]. Another group used a different severe SMA mouse model to show that selective expression of SMN in MNs only partially rescues the model, with little effect on survival [61]. When both central nervous system and muscle-specific inductions of SMN expression are combined, there is a much more successful rescue of the SMA phenotype than with expression in either tissue alone [60], [62]–[65]. However, the earliest symptoms of the disease are muscle weakness and muscle atrophy, with only about 15–35% MNs lost in the severe SMA I patients [17]. SMN protein, the loss of which underlies SMA, is expressed throughout the body, including spinal cord and skeletal muscle [20], [66], and is required for fetal development in mice [43], [56], [57], [67], [68]. It is therefore reasonable to assume that SMN has important functions in multiple tissues, and may affect spinal cord and skeletal muscle independently.

SMN knockdown in the C2C12 mouse myoblast cell line results in myotube maturation defects [61], suggesting a MN-independent muscle disease in SMA. Our findings support those of Mutsaers et al., who showed severe muscle degeneration in a fully innervated muscle group (LAL), with no concomitant MN pathology in a severe SMA mouse model at P1 [69]. This group also found upregulation of proteins associated with apoptosis and myopathies, including the early DNA damage response protein γH2AX [54]. These results and our findings argue that muscle disease in SMA is initiated prenatally and independently of MN disease, and that SMA results in DNA damage, apoptosis, and denervation-independent muscle degeneration.

Most studies of SMN function have investigated its role in spliceosomal assembly and RNA splicing [70]–[76], but more recent work has revealed additional functions for SMN. SMN is located in the cytoplasm and nucleus of cells [72]. Dreyfuss and colleagues discovered that SMN is necessary for the assembly of the cytoplasmic Sm core [70], [73], [76]. In the nucleus, SMN is concentrated in structures called Gemini of coiled bodies (or “gems”) that function in snRNP biogenesis [72]. SMN interacts directly with several Gemin proteins and Sm proteins [71], [76]. The SMN-Gemin2 interaction forms part of the SMN complex that promotes assembly of spliceosomal snRNPs [75]. More recent studies have found that the human SMN-Gemin2 complex also interacts with RAD51. The Gemin2-RAD51 complex may facilitate repair of DNA double-strand breaks by homologous recombination [77], [78]. Our work showing prominent DNA damage accumulation in skeletal muscle of SMN mice indicates possible DNA repair-related functions of SMN.

Based on this newly discovered putative relationship between SMN and DNA repair, we hypothesized that DNA damage accumulation is part of the pathobiology of SMA in vivo. Both the TUNEL method and the Southern blot method we used employ the TdT enzyme. TdT adds nucleotides to the 3′ overhang or blunt ends of double-strand breaks in DNA [50]. The TUNEL method has also been widely used as a cellular assay for apoptosis [45], [79]. The Southern blot allowed us to detect internucleosomal fragmentation indicated by a laddering of DNA on gel electrophoresis. Both the TUNEL method and TdT-based Southern blotting are widely accepted methods of detecting DNA damage [44], [50], [80]. Our results, therefore, show both cell death and DNA damage. To differentiate between the two processes, future studies must investigate which cell death pathways are activated in SMA, when they become activated in relation to the emergence of DNA fragmentation, and whether SMN-depleted cells have defective DNA repair.

Our observations of prenatal DNA damage in SMA skeletal muscle support the view of SMA as an embryonic disease of skeletal muscle. The histological TUNEL results at E13 and E15.5 did not reveal statistically significant increases in DNA damage in SMA skeletal muscle or spinal cord. TUNEL counts are slightly elevated in both SMA ventral horn and skeletal muscle at E13. There is evidence that SMA is an embryonic disease: SMN knockout is embryonic lethal in mice [43], [56], [57], [68], and babies with severe SMA exhibit reduced fetal movements, bone fractures, and muscle contractures at birth [2], [81]. If cell death occurs during embryonic development after E15.5, P5 may be too late to identify dying cells in the spinal cord. It will be informative to perform TUNEL staining at later embryonic time points in SMA mice to identify the developmental stage when most cell death occurs.

In the studies described here, we chose to quantify cell death in the ventral horn of the spinal cord because this is where MNs are located. MN numbers in the lumbar and cervical spinal cord are comparable between SMA and control mice at P5 (Figure 6A, 6C) and E15.5 (Figure 6B, 6D). There are no TUNEL-positive nuclei colocalized with ChAT-positive MNs, indicating that the few TUNEL-positive cells in the ventral horn are not MNs. Thus, we have not observed a loss of spinal cord MNs or ongoing cell death in MNs in this SMA mouse model. However, we do not know whether the observed surviving MNs are functioning properly and making appropriate connections with muscle targets. It is also possible that MNs may be lost at a later stage in disease in these SMA mice, after cell death in skeletal muscle has commenced.

In contrast to the paucity of TUNEL-positive profiles in spinal cord, the increase in TUNEL signal in postnatal mouse SMA skeletal muscle is widespread. Several groups have reported selective denervation of specific skeletal muscle groups in SMA mice, with other muscle groups spared [55]. Of the hindlimb muscle groups in which we found cell death, soleus has been reported as still innervated in the “Burghes” SMA mouse model at P13 [34], and soleus, TA, MG/LG, and Edl have been found to be still innervated in the “Taiwanese” SMA mice at P5 and P9 (personal communication from Te-Lin Lin, laboratory of Yuh-Jyh Jong, Kaohsiung Medical University, Taiwan). The presence of cell death in muscle groups that are still innervated suggests that muscle degeneration in SMA is independent of MN degeneration and is a primary disease mechanism. However, disease may also be initiated at the NMJ, as several studies have suggested [23], [33], [82], [83]. It is not known whether skeletal muscle disease in SMA is independent of the presynaptic component of the NMJ.

Despite widespread skeletal muscle cell death and smaller muscle size, SMA mouse muscle appears to have normal tissue anatomy, with normal myofiber morphology and all muscle groups represented (Figure 1A). SMA myofiber diameters and density (per muscle area) are comparable to control (Figure 2D and 2E). Thus, the SMA mice described here appear to have fewer total myofibers, with preserved myofiber size and density. These findings agree with those of Ling et al. in the “delta 7” SMA mouse model [84]. This group reported that twitch tension induced by nerve and muscle stimulation was comparable to controls, but tetanic force was smaller, which could be explained by the smaller total size of the muscle in SMA mice. Thus, the muscle weakness which is a hallmark of SMA could be explained by smaller total muscle mass, rather than a dysfunction of the muscle contractile apparatus or faulty transmission across the NMJ. However, these results do not explain why SMA muscles are smaller. RNA microarray for cell death and DNA repair pathway activation did not uncover any differences between skeletal muscle from SMA mice and control littermates. Because the array did not cover all apoptotic pathways, it is possible that we missed a pathway not involved in DNA damage-induced apoptosis, such as the caspase-independent AIF-induced mechanism [85]. Alternatively, the lack of global changes in expression may be due to selective vulnerability of satellite cells or subtypes of myofibers and the asynchronous cell death in these cells [26], [36]. Our EM analysis shows evidence of satellite cell apoptosis. If satellite cells are dying in developing SMA muscle, it is possible that the muscle will have smaller growth capacity over time, resulting in delayed expansion of the mature myotube population. Such muscles would have normal myofiber size and density, but a smaller total myofiber number [84], which would increasingly lag behind that of control littermates as development progressed. This phenomenon is what we have observed in the present report: a sustained lag in weight gain in SMA mice compared to control littermates and smaller but otherwise morphologically normal muscles (Figure 2). Furthermore, the TUNEL-positive cells in muscle are distributed throughout the muscle, and apoptotic cells appear to be surrounded by other healthy myocytes. These findings argue against grouped atrophy or a muscular dystrophy-like phenotype of fulminant necrosis [86]. Rather, the pathological process in SMA mouse muscle is more subtle, distributed, and asynchronous. Satellite cell defects have been reported in human SMA [25], [35]. Our finding that Pax-positive satellite cells can be TUNEL-positive in SMA mice, and that satellite cells are dying by classical apoptosis identifies a possible satellite cell defect and warrants further exploration of muscle satellite cell pathobiology in SMA mouse models. A useful experiment would be to block apoptosis specifically in skeletal muscle to see if this extends survival.

The excessive DNA damage accumulation that we report in embryonic SMA muscle is a newly observed pathological feature of SMA in mice, perhaps signifying increased DNA fragility or faulty DNA repair. The mechanism of action by which SMN causes SMA remains unknown. We investigated DNA damage in SMA because several publications have linked SMN with DNA repair. SMN and its binding partner, Gemin2, have been shown to interact with the DNA repair protein RAD-51 [78] and facilitate DNA repair [77]. SMN also competes for binding sites of coilin with DNA repair proteins ku70 and ku80 [87]. Unlike MNs, skeletal muscle undergoes multiple cell divisions throughout development and adulthood, making it more susceptible to DNA replication-repair defects. Such defects would disproportionately affect dividing skeletal muscle satellite cells, inhibiting normal myofiber expansion during development and regeneration, and resulting in smaller muscle mass. Faulty DNA repair would also decrease the regenerative potential of muscle undergoing intrinsic disease or denervation. Both retarded muscle growth and denervation are important components of human SMA disease progression, and thus more studies are needed to investigate whether SMA myocytes have an intrinsic defect in differentiation or DNA repair. Several studies have already suggested that SMA myocytes have delayed maturation and defective myotube fusion [37], [88].

Our findings of early cell death in the skeletal muscle of SMA mice support the need for early treatment intervention in SMA. An intrinsic muscle defect in SMA would provide exciting treatment opportunities targeting myocyte survival and differentiation.

## Supporting Information

Figure S1 **Extensive cell death in P5 SMA skeletal muscle (LG, MG, and Sol).** TUNEL (green) was performed on transverse sections of the lower hindlimb at P5. Hoescht (blue) was used to stain cell nuclei. The red channel was used to exclude autofluorescent signal (e.g. red blood cells) from analysis. Sol – soleus muscle, LG – gastrocnemius lateralis muscle, MG – gastrocnemius medialis muscle.
(TIF)

Figure S2 **Western blots for DNA damage response proteins confirm gene array results.** Immunoblotting was performed on homogenates from whole leg skeletal muscle and whole spinal cords of SMA mice and control littermates at postnatal day 3. Blots were analyzed using Image J, with protein-specific bands normalized to Ponceau bands in the same size range of the same blot to control for protein loading. Bar graphs show Mean ± SD of the two bands per group shown above. Molecular weights (kD) are shown on the right side.
(TIF)

Table S1 **Complete list of genes included in the DNA damage signaling gene array.** A commercially available gene array (SABiosciences array PAMM-029ZD-12) was used to assay gene expression in skeletal muscle. The array included 84 genes associated with DNA damage detection, DNA repair, apoptosis, cell cycle, and other functions.
(DOC)

File S1 **Videos S1–S7: Videos of muscle weakness in SMA mouse.** Videos of an SMA mouse and its control littermate show muscle weakness and weight loss on postnatal days 6 (P6) and 9 (P9). Video S1– control mouse at P6, suspension test. Video S2– SMA mouse at P6, suspension test. Video S3– control and SMA mice at P6, rollover test. Video S4– control mouse at P9, suspension test. Video S5– control mouse at P9, suspension test. Video S6– control mouse at P9, rollover test. Video S7– SMA mouse at P9, rollover test.
(ZIP)
