# Supplementary figures and images for: Skeletal Muscle DNA Damage Precedes Spinal Motor Neuron DNA Damage in a Mouse Model of Spinal Muscular Atrophy (SMA)
Source: PLoS One. 2014 Mar 25;9(3):e93329. doi: 10.1371/journal.pone.0093329 (PMC3965546; doi:10.1371/journal.pone.0093329)

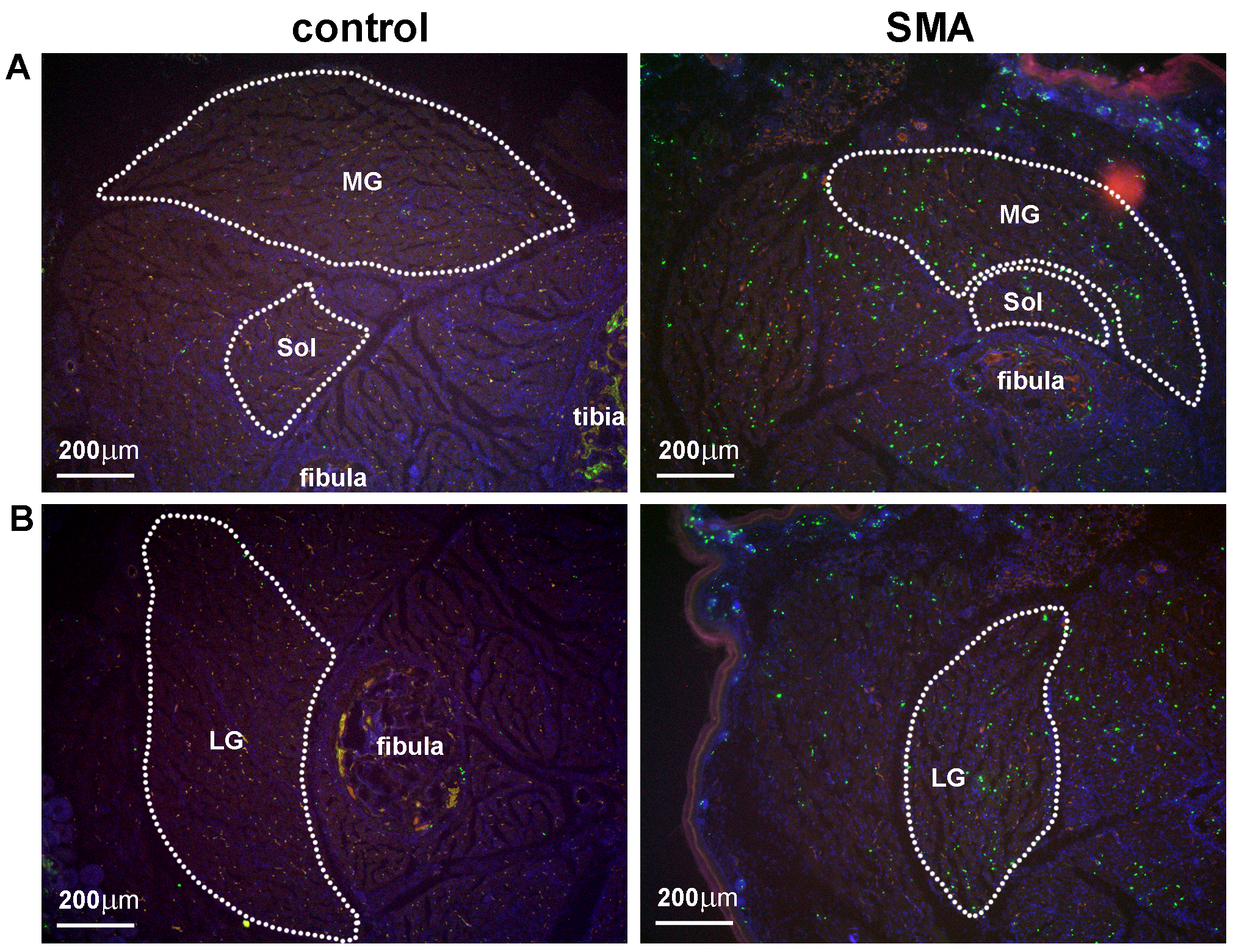

Supplement: Figure S1 — Extensive cell death in P5 SMA skeletal muscle (LG, MG, and Sol). TUNEL (green) was performed on transverse sections of the lower hindlimb at P5. Hoescht (blue) was used to stain cell nuclei. The red channel was used to exclude autofluorescent signal (e.g. red blood cells) from analysis. Sol – soleus muscle, LG – gastrocnemius lateralis muscle, MG – gastrocnemius medialis muscle. (TIF) [file pone.0093329.s001.tif]

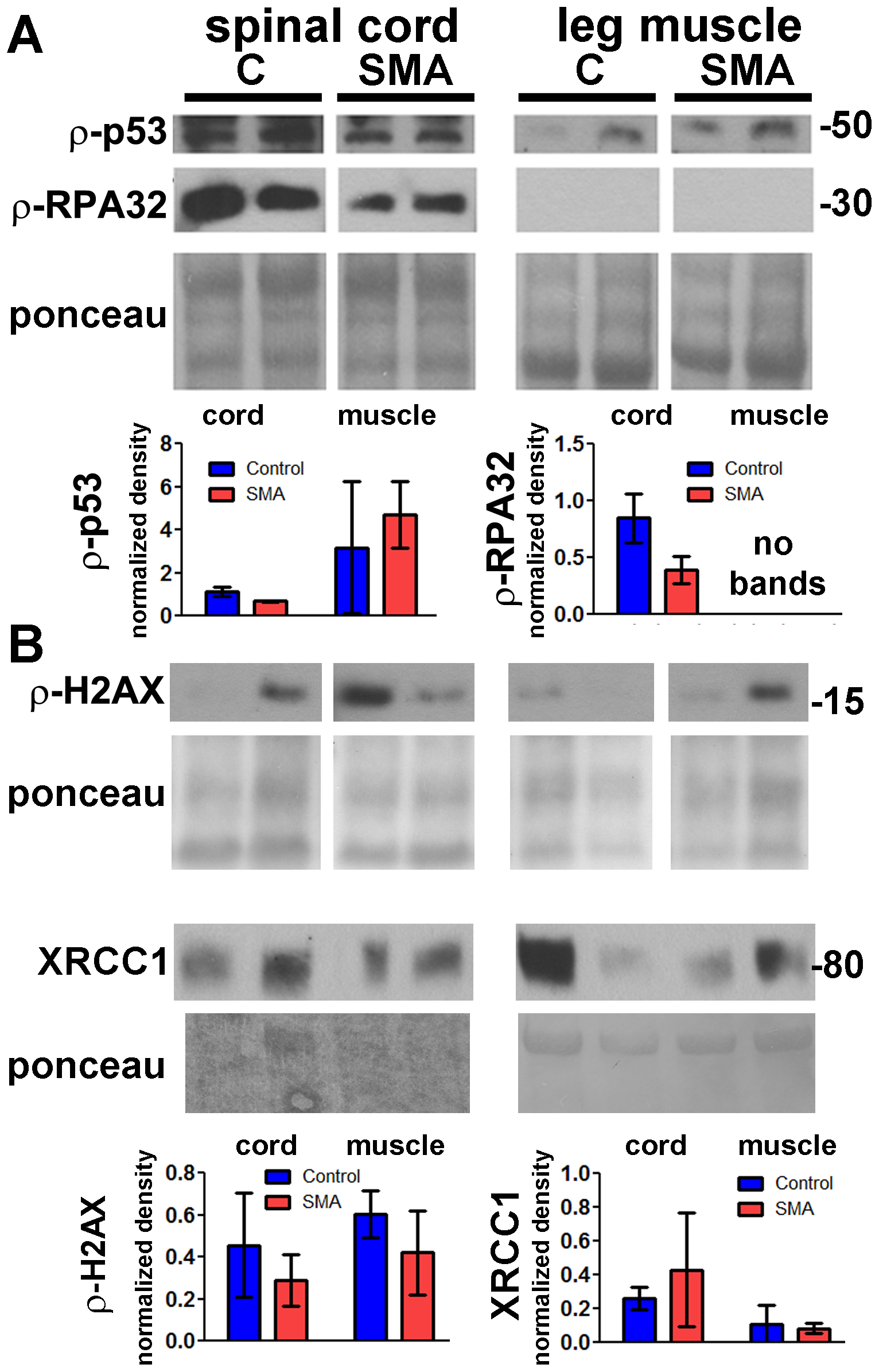

Supplement: Figure S2 — Western blots for DNA damage response proteins confirm gene array results. Immunoblotting was performed on homogenates from whole leg skeletal muscle and whole spinal cords of SMA mice and control littermates at postnatal day 3. Blots were analyzed using Image J, with protein-specific bands normalized to Ponceau bands in the same size range of the same blot to control for protein loading. Bar graphs show Mean ± SD of the two bands per group shown above. Molecular weights (kD) are shown on the right side. (TIF) [file pone.0093329.s002.tif]
