# Supplementary material for: Skeletal Muscle DNA Damage Precedes Spinal Motor Neuron DNA Damage in a Mouse Model of Spinal Muscular Atrophy (SMA)
Source: PLoS One. 2014 Mar 25;9(3):e93329. doi: 10.1371/journal.pone.0093329 (PMC3965546; doi:10.1371/journal.pone.0093329)
Supplement: Table S1 — Complete list of genes included in the DNA damage signaling gene array. A commercially available gene array (SABiosciences array PAMM-029ZD-12) was used to assay gene expression in skeletal muscle. The array included 84 genes associated with DNA damage detection, DNA repair, apoptosis, cell cycle, and other functions. (DOC) [file pone.0093329.s003.doc]

| **Symbol** | **RefSeq** | **Symbol** | **RefSeq** | **Symbol** | **RefSeq** |
| --- | --- | --- | --- | --- | --- |
| Abl1 | NM_009594 | Lig1 | NM_010715 | Rad50 | NM_009012 |
| Apex1 | NM_009687 | Mbd4 | NM_010774 | Rad51 | NM_011234 |
| Atm | NM_007499 | Mcph1 | NM_173189 | Rad51c | NM_053269 |
| Atr | NM_019864 | Mdc1 | NM_001010833 | Rad51l1 | NM_009014 |
| Atrx | NM_009530 | Mgmt | NM_008598 | Rad52 | NM_011236 |
| Bax | NM_007527 | Mif | NM_010798 | Rad9 | NM_011237 |
| Blm | NM_007550 | Mlh1 | NM_026810 | Rev1 | NM_019570 |
| Brca1 | NM_009764 | Mlh3 | NM_175337 | Rnf8 | NM_021419 |
| Brca2 | NM_009765 | Mpg | NM_010822 | Rpa1 | NM_026653 |
| Brip1 | NM_178309 | Mre11a | NM_018736 | Smc1a | NM_019710 |
| Cdc25a | NM_007658 | Msh2 | NM_008628 | Smc3 | NM_007790 |
| Cdc25c | NM_009860 | Msh3 | NM_010829 | Sumo1 | NM_009460 |
| Cdkn1a | NM_007669 | Nbn | NM_013752 | Terf1 | NM_009352 |
| Chek1 | NM_007691 | Nthl1 | NM_008743 | Topbp1 | NM_176979 |
| Chek2 | NM_016681 | Ogg1 | NM_010957 | Trp53 | NM_011640 |
| Dclre1a | NM_018831 | Parp1 | NM_007415 | Trp53bp1 | NM_013735 |
| Ddb2 | NM_028119 | Parp2 | NM_009632 | Ung | NM_011677 |
| Ddit3 | NM_007837 | Pcna | NM_011045 | Wrn | NM_011721 |
| Ercc1 | NM_007948 | Pms2 | NM_008886 | Xpa | NM_011728 |
| Ercc2 | NM_007949 | Pole | NM_011132 | Xpc | NM_009531 |
| Exo1 | NM_012012 | Polh | NM_030715 | Xrcc1 | NM_009532 |
| Fanca | NM_016925 | Poli | NM_011972 | Xrcc2 | NM_020570 |
| Fancc | NM_007985 | Ppm1d | NM_016910 | Xrcc3 | NM_028875 |
| Fancd2 | NM_001033244 | Ppp1r15a | NM_008654 | Xrcc6 | NM_010247 |
| Fancg | NM_053081 | Prkdc | NM_011159 | Actb | NM_007393 |
| Fen1 | NM_007999 | Pttg1 | NM_013917 | B2m | NM_009735 |
| Gadd45a | NM_007836 | Rad1 | NM_011232 | Gapdh | NM_008084 |
| Gadd45g | NM_011817 | Rad17 | NM_011233 | Gusb | NM_010368 |
| H2afx | NM_010436 | Rad18 | NM_021385 | Hsp90ab1 | NM_008302 |
| Hus1 | NM_008316 | Rad21 | NM_009009 |  |  |
